# Predicting Future Driving Risk of Crash-Involved Drivers Based on a Systematic Machine Learning Framework

**DOI:** 10.3390/ijerph16030334

**Published:** 2019-01-25

**Authors:** Chen Wang, Lin Liu, Chengcheng Xu, Weitao Lv

**Affiliations:** 1 Jiangsu Key Laboratory of Urban ITS, Southeast University, Nanjing 210096, China; wkobec@hotmail.com; 2 Intelligent Transportation Research Center, Southeast University, Nanjing 210096, China; 3 Jiangsu Intelligent Transportation Systems Co., Ltd., Nanjing 210096, China; lin.liu@jiangsuits.com (L.L.); lwt@jiangsuits.com (W.L.)

**Keywords:** driving risk, traffic violation behavior, machine learning, temporal transferability

## Abstract

The objective of this paper is to predict the future driving risk of crash-involved drivers in Kunshan, China. A systematic machine learning framework is proposed to deal with three critical technical issues: 1. defining driving risk; 2. developing risky driving factors; 3. developing a reliable and explicable machine learning model. High-risk (HR) and low-risk (LR) drivers were defined by five different scenarios. A number of features were extracted from seven-year crash/violation records. Drivers’ two-year prior crash/violation information was used to predict their driving risk in the subsequent two years. Using a one-year rolling time window, prediction models were developed for four consecutive time periods: 2013–2014, 2014–2015, 2015–2016, and 2016–2017. Four tree-based ensemble learning techniques were attempted, including random forest (RF), Adaboost with decision tree, gradient boosting decision tree (GBDT), and extreme gradient boosting decision tree (XGboost). A temporal transferability test and a follow-up study were applied to validate the trained models. The best scenario defining driving risk was multi-dimensional, encompassing crash recurrence, severity, and fault commitment. GBDT appeared to be the best model choice across all time periods, with an acceptable average precision (AP) of 0.68 on the most recent datasets (i.e., 2016–2017). Seven of nine top features were related to risky driving behaviors, which presented non-linear relationships with driving risk. Model transferability held within relatively short time intervals (1–2 years). Appropriate risk definition, complicated violation/crash features, and advanced machine learning techniques need to be considered for risk prediction task. The proposed machine learning approach is promising, so that safety interventions can be launched more effectively.

## 1. Introduction

Since 2018, the Kunshan Traffic Police Department in China has been developing a comprehensive safety improvement plan, aiming to control the increasing numbers of crashes. Limited by resources, the policy-makers intended to focus on specific driver groups with high driving risk instead of all registered drivers. Drivers with prior crash involvement were considered as a starting point, since they were often considered at-risk and had been largely examined by previous literature [1,2,3]. The driving risk of those drivers was expected to be accurately predicted for a future short time period (e.g., one or two years). As such, safety interventions can be implemented in a more effective and efficient way.

This practical need has raised several technical issues. First, how to define driving risk? According to the local government, only crash-involved drivers were considered at the first stage of the plan. Thus, latent risk, such as near-miss events [4], were not considered. Moreover, driving risk was defined from multiple aspects, such as crash severity, crash recurrence, and fault commitment. For example, some researchers defined high-risk drivers as those who were involved in crashes more than expected [5,6]. At-fault drivers were also considered to be risky and have been extensively studied [7,8,9,10,11]. Crash severity was another dimension of research interest [12,13,14,15].

Determining what factors should be used for predicting driving risk is the second issue. Previous literature has extensively examined various driver factors, including risky driving behaviors, psychological factors, demographics, and social-economic factors [15,16]. Among them, risky driving behaviors were often considered as important factors correlated to crash outcomes [17,18]. Previous literature largely used prior violation/crash factors to represent risky driving behaviors. For example, Gebers et al. [19] used prior violation frequency and crash frequency as crash risk predictors. Buckley et al. [12] predicted the future injury risk for adolescents, considering their prior violation frequency. Zhang et al. [13] linked risky driving behavior factors to injury risk, by developing a factor of prior violation frequency. No type-specific violation factors were considered, as claimed by the authors. Nishida [20] found that elderly drivers with prior cell phone usage violation had higher fatal accident ratios. Hosseinlou et al. [21] attempted to link driving violations to crash risk at an aggregated level. Three violation types were found as significant, including illegal overtaking, tailgating, and speed violation.

The third issue is finding a way to precisely capture the driving risk pattern. Previous literature largely depended on traditional statistical methods, including logistic regression [7,11,22], canonical correlation [19], Poisson/negative binomial regression [12,20,23]. As for prediction performance, Gebers et al. [19] reported a relatively low prediction precision of 27.2% on drivers’ future crash involvement. The authors pointed out the weakness of the statistical model of considering simple interactions between variables and outcomes. Das et al. [11] reported 62% precision of drivers’ future fault assignment, based on a logistic model.

Although previous literature has extensively studied the above issues, some gaps still need to be further addressed. First of all, most previous studies focused on a certain perspective (e.g., severity) of driving risk, instead of comprehensively investigating it from multiple dimensions. Second, as for predictors, a limited number of risky driving factors were examined based on historical violation/crash records. More factors need to be examined, such as penalty fees, penalty points, and the temporal and spatial characteristics of prior violations records. Those could also reflect risky driving behaviors. Third, regarding risk pattern identification, previous models generally reported low predicting accuracy. It appeared that the risk pattern should be captured in a more complexed way. Also, a model validation process was missing in most studies. To fill the gaps, this study proposed a systematic machine learning approach to predict future driving risk of crash-involved drivers in Kunshan, China. First, driving risk was defined in different ways, considering multiple dimensions. Then, a number of risky driving factors were carefully developed based on violation/crash records. Finally, multiple tree-based machine learning models were developed to capture the complex driving risk pattern, and further validated by transferability tests.

## 2. Materials and Study Design

### 2.1. Data

The Kunshan Traffic Police Department provided seven-year (2011–2017) crash data, including crash records, crash characteristics, vehicle characteristics, roadway conditions, and drivers’ demographic information (i.e., gender, age, driving age, and so on) can be collected. Additionally, on-site traffic violation records from 2011–2017 were also acquired from the Kunshan Traffic Police Department. By matching IDs in the crash database and violation database, drivers’ violation records were also collected. Excluding those records with incomplete information, 201,328 crash records were kept and a total of 387,836 drivers were observed. All these drivers were used as instances (i.e., samples) for the machine learning model development.

### 2.2. Study Design

**Figure 1:**
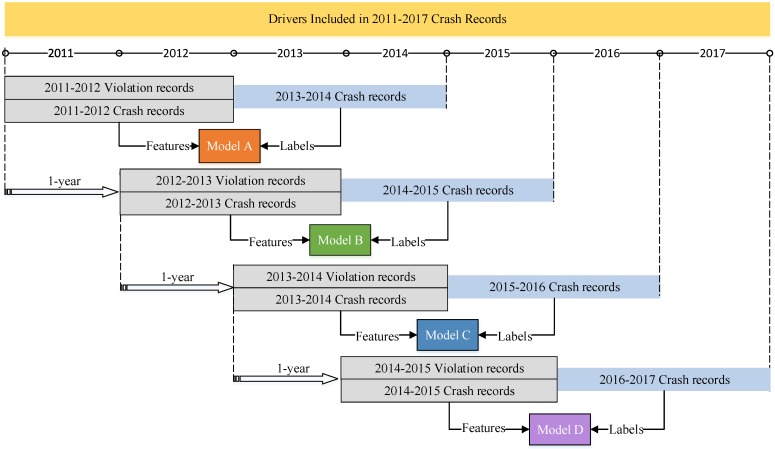
Data usage for model development with a one-year sliding time window.

Regarding the request from the Kunshan Police Department, prediction models are expected to be robust and transferable. Thus, prediction models for four consecutive time-periods were developed in order to examine temporal transferability. As shown in Figure 1, the basic idea of the model fitting process is to use drivers’ two-year prior crash/violation records (i.e., prior features) to predict their future driving risk (i.e., labels) in subsequent two years. For example, the first model (i.e., Model A) was established to predict drivers’ driving risk during the 2013–2014 time period, using their prior two-year (2011–2012) violation/crash records. Using a one-year sliding time window, prediction models for 2014–2015 (i.e., Model B), 2015–2016 (i.e., Model C), and 2016–2017 (i.e., Model D) can be developed. 

## 3. Model Development and Analysis

### 3.1. Model Framework

**Figure 2:**
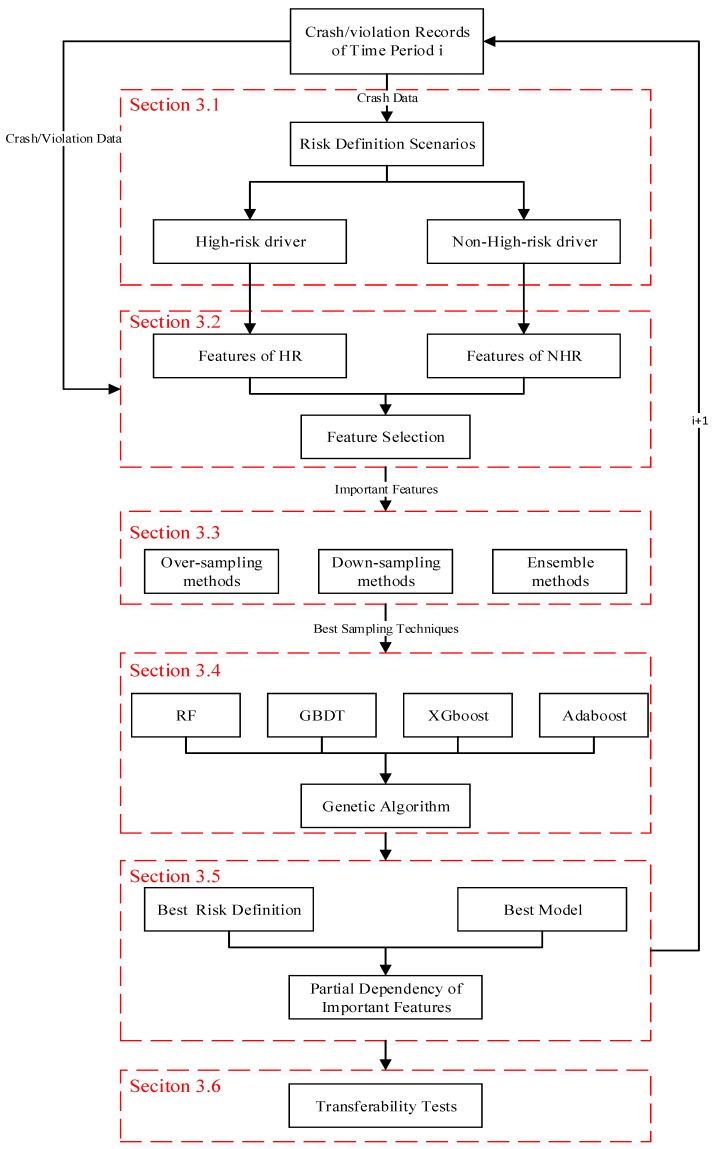
A general framework of model development and analysis.

The model development of different time periods (i.e., Model A to D) followed the same procedure. A systematic machine learning framework was proposed, as shown in Figure 2. The details are discussed in the following sub-sections.

### 3.2. Driving Risk Definition

Based on previous studies, recurrent crash involvement and fault assignment were considered as the two important aspects for defining high-risk drivers. According to the local traffic police, high-crash-risk drivers were considered to meet two criteria: 1. they have recurrent crashes within a short time period; 2. they have found to be at-fault in severe crashes. The first criterion is consistent with many previous studies, relying on the knowledge that aggressive/careless drivers may be more likely to be involved in crashes. Since our observation time is two years, drivers with recurrent crashes (i.e., >2) within this period were considered to meet this criterion. In other words, drivers with two or more crashes during a two-year period may be considered as high-risk. The second criterion is slightly different from previous literature. In China, there are five levels of fault conviction for drivers: none, minor, equal, major, and full. In practice, there are some conditions that drivers will be assigned a fault without aberrant behaviors, especially in non-severe crashes. Non-severe crashes refer to crashes without any injuries. Severe crashes include injury and fatal crashes. The Kunshan traffic police claimed that at-fault assignment in severe crashes is much more reliable than that in non-severe crashes. Based on this knowledge, five scenarios were used to define high-risk (HR) drivers in Kunshan: 1. drivers with recurrent crash involvement; 2. drivers determined as at-fault in severe crashes; 3. drivers with recurrent crash involvement or found as at-fault in severe crashes; 4. drivers with at-fault crash involvement; 5. drivers with crash involvement. For each scenario, excluding HR drivers, the remaining drivers were defined as non-high-risk (NHR) drivers. It should be noted that the driving risk of a driver varies over time. In other words, one can be defined as HR in one observation period (e.g., 2016–2017) but NHR in another period (e.g., 2014–2015), based on his/her crash records. Table 1 shows the basic statistics of HR and NHR driver group under the three scenarios in different time periods.

### 3.3. Feature Extraction and Selection

#### 3.3.1. Feature Extraction

Feature engineering is an important process of extracting and selecting proper features for machine learning model development. For each time slice, drivers’ demographic information can be easily collected as candidate features. In this study, drivers’ gender (male/female), car type ownership (small/medium/large passenger car, small/large truck, bus, and others), occupation (i.e., employee, free-lancer, farmer, student, employer, government officer, and unemployed person), and nationality (China/Foreign) were extracted to develop features. Regarding age, drivers were classified into three groups: young drivers (<30), middle-aged driver (<65), and older driver (>65). Driving experience refers to how many years one has been driving. Note that age and driving experience are time-varying variables.

Risk driving features were developed based on traffic violation and crash records. The important step is to develop features describing risky driving behaviors. Traffic violation and crash records were the sources. Traffic violation is well-known to be correlated with increased accident propensity and reflects both risk-taking, social nonconformity, and exposure. Researchers have used past violation records as predictors for drivers’ subsequent crash involvement. Although some claimed that they were not as effective as expected [24], it should be known that some also found the relationship between violation records and driver risk [19,22,25]. For example, Stamatiadis et al. [26] found that 2.1% of licensed drivers who are charged with six or more points in the past two years accounted for 5.3% of all crashes. Additionally, traffic violations were largely found for Chinese drivers [27]. Thus, features selection based on traffic violation records was considered to be crucial. First of all, violation frequency was considered to indicate the overall aberrant behavior of a driver. Two features were developed: cumulative violation frequency and cumulative violation types. Records of violation penalty points and violation penalty fee were used to develop several important features: maximum one-time violation penalty fee/points, cumulative violation penalty fee/points, and average violation penalty fee/points (per time). Besides these indicating the overall violation condition of a driver, more detailed features were also extracted. First, certain types of violation records were considered useful to unveil detailed driving behavior. However, there are over 200 traffic violations types found in the database and it is unnecessary to incorporate all those features in the model development process. Thus, the top 80 violation types (ranked in frequency) were used for feature extraction from violation records. The reasons are two-fold: 1. they were all counted over 10 times per year, suggested as the minimum sample size for parametric statistics tests by previous literature [28]; 2. they accounted for over 99% of all violation counts. Second, temporal violation features were developed. For example, if one has two prior violation records during the morning peak-hour traffic in Kunshan (i.e., 7–9 am), the feature value for morning peak-hour violation (MPHV) will be coded as 2. Third, spatial violation features were also considered. All violation locations were classified into 4 groups based on their prior crash records, based on total crash frequency and severe crash frequency. Type 1 (TP1) indicates a location with a high (i.e., more than average) number of total crash and high severe crash frequency. Type 2 (TP2) indicates a location with high crash frequency but less severe crashes. Type 3 (TP3) describes a location with low crash frequency but high severe crashes. Type 4 (TP4) encompasses locations with low and severe crash frequencies. If one has three prior violations at TP 4 locations, then the feature value of TP4 will be coded as 3 for this driver. Based on crash records, several features were also developed to describe drivers, in terms of their overall crash involvement, fault assignment, and specific crash type involvement. For crash involvement, two features are cumulative crash involvement and cumulative severe crash involvement. Regarding fault assignment, at fault crash and severe at-fault crash were considered. Crash type I was developed to describe crashes between vehicles (e.g., rear-end, head-on, single crash etc.), while crash type II was used to indicate crashes between vehicles and other road users. Drivers’ prior records of intoxicated driving were also used to develop a feature.

#### 3.3.2. Feature Selection

There are totally 125 features extracted from prior crash/violation records. Then, a feature selection procedure was applied based on the random forest (RF) technique. RF was often used to rank feature importance so that non-important features can be discarded. The feature importance of the RF model was examined using the Gini index, which can be calculated as follows:

(1) $$G=\sum_{c=1}^{C}P_{j}^{m}\left(1-P_{j}^{m}\right)$$

(2) $$P_{j}^{m}=\frac{1}{N^{m}}\sum_{x_{i}^{m}}I\left(y_{i}^{m}=c\right)$$

where

$P_{j}^{m}$: Proportion of class *c* observations in node *m*

$N^{m}$: Number of observations received at node *m*

$y_{i}^{m}$: the response value corresponding to the observation *i* at node *m*

$x_{i}^{m}$: the feature vector corresponding to the observation *i* at node *m*

*c:* class

*C:* the total number of classes

For the 2016–2017 model development, the top 50 features were finally selected based on their relatively larger Gini index. Note that for other time periods, the order of top features was slightly different. This could be considered as reasonable based on the assumption that drivers’ risk pattern tends to be time-varying. In general, top features were consistent across four observation periods. Table 2 presents final features extracted for 2016–17 model development (i.e., Model D) based on RF.

### 3.4. Sampling Techniques

**Figure 3:**
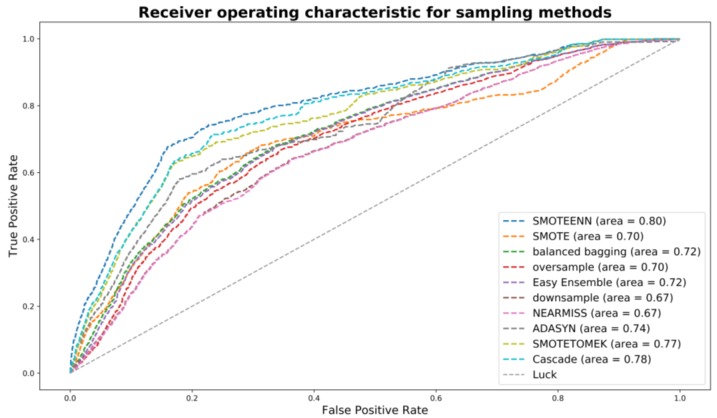
Receiver operating characteristic (ROC) curves of all sampling methods for 2016–2017 periods (scenario 3).

In this study, datasets were unbalanced by the limited number of high-risk drivers, which could cause model overfitting. Thus, imbalanced sampling techniques were introduced to deal with the issue. Multiple sampling techniques [29] were examined, including random down-sampling, near-miss down-sampling (type 1, 2, and 3), adaptive synthetic sampling approach for imbalanced learning (ADASYN), random minority over-sampling with replacement, SMOTE (borderline and regular), balance cascade sampling, balanced bagging classifier with random forest, easy ensemble sampling, SMOTE-ENN, and SMOTE-Tomek. ADASYN, SMOTE, and random over-sampling are three common over-sampling methods, which use nearest neighbors to construct synthetic samples. Near-miss and random down-sampling are two commonly used down-sampling methods. Balance cascade sampling, balanced bagging classifier with random forest, and easy ensemble sampling are three ensemble sampling methods. The core idea of balance cascade sampling and easy ensemble sampling is to create an ensemble of balanced sub-datasets by iteratively under-sampling the imbalanced dataset using an estimator (i.e., random forest). As for balance cascade sampling, the K-neighbors classifier estimator was used. As for easy ensemble, Adaboost estimator was used as the base estimator. Balanced bagging classifier is a combination method of bagging and random down-sampling to create balanced training sets for classifiers. SMOTE-ENN and SMOTE-Tomek are two sampling methods combining both over- and under- samplings. Both were examined based on the random forest as a base estimator. The receiver operating characteristic (ROC) curves of all sampling methods for 2016–2017 periods (scenario 3) are shown in Figure 3. 

According to the results, SMOTE-ENN generally appeared to be better than other sampling techniques, in terms of achieving higher AUC. The ROC curves of all sampling methods for 2016–2017 periods (scenario 3) are shown in Figure 3. To further improve model performance, a genetic algorithm (GA) and multiple machine learning models were applied with SMOTE+ENN sampling method, which will be discussed in the subsequent sections. 

### 3.5. Model Training with Genetic Algorithm

Based on SMOTE+ENN sampling techniques, four machine learning techniques were utilized to discriminate HR drivers from NHR drivers, including random forest (RF), extreme gradient boosting decision tree (XGboost), gradient boosting decision tree (GBDT), and Adaboost with decision tree as the classifier. They are all ensemble learning techniques based on decision trees. The basic ideas of two ensemble methods are briefly discussed here including bagging and boosting. Bagging randomly selects samples from a training dataset with a replacement (i.e., bootstrapping) and fits models on each random sample. The process is repeated a number of times and results are predicted with the average of each of the fitted models. Similar to bagging, boosting also utilizes bootstrap to randomly select samples from the training set. The major difference is that boosting focuses on dealing with cases which were too difficult to be handled by previous fitted models. For example, Adaboost is a typical boosting strategy that improves model performance by iteratively assigning increased weights to those positive samples, which were wrongly classified by previous models. In general, RF belongs to the bagging ensemble learning, while GBDT, XGboost, and Adaboost belong to boosting ensemble methods. A detailed model algorithm was not discussed here and can be found in Hastie et al. [30].

**Figure 4:**
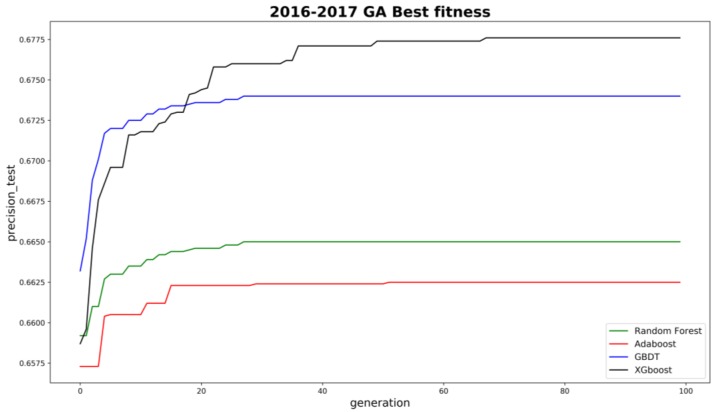
Convergence of four machine learning models by GA hyperparameter tuning.

As for model parameters tuning, the grid search method was normally used to find optimal hyperparameters for machine learning models. However, it is time-consuming and impractical to tune models like XGboost with numerous hyperparameters. In order to automatically find the best hyperparameters for each machine learning model, a genetic algorithm (GA) was utilized. The fitness function was set as the average precision (AP) on training sets, with 10-fold cross-validation. In this study, the positive samples are limited so AP could be more appropriate than AUC [31]. A parallel computing technique was also introduced to boost model convergence. Thanks to the GA, all model performances have significantly improved compared to default hyperparameters. Figure 4 shows the convergence results for the four models for model D development.

### 3.6. Model Performance Analysis

#### 3.6.1. Comparison of High-Risk Labeling Scenarios 

**Figure 5:**
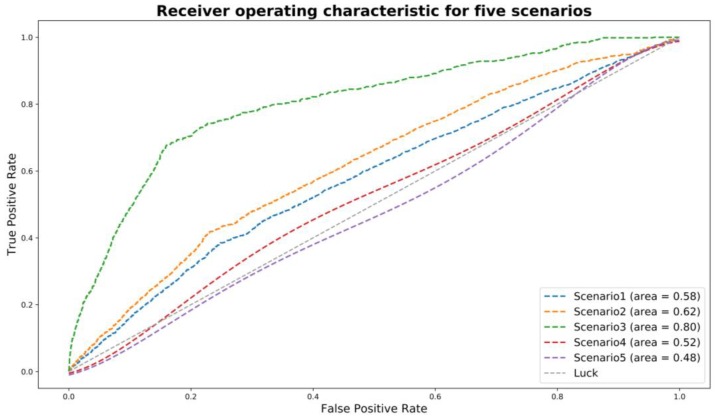
ROC curve for five labeling scenarios for model D development.

Five scenarios were all examined. Scenario 3 appears to be better than the other four scenarios, by achieving the best overall model performance in both training and testing datasets. Figure 5 presents the ROC curves for five scenarios for the best model for 2016–2017 period (i.e., model D). As claimed by the local police, the fault assignment in non-severe crashes was not as reliable as that in severe crashes. Thus, it is reasonable that scenario 4 had a lower AUC of 0.52. That is, there could be many cases where drivers at-fault were actually not at-fault. Scenario 5 did not perform well since they did not consider fault. As reported in previous literature, fault and non-fault drivers were significantly different in many aspects. Scenario 1 and scenario 2 are subsets of scenario 3. The lower AUC of those two scenarios and a higher AUC of scenario 3 indicate that fault assignment and recurrent crash involvement could be both important to identify driving risk. Ideally, if fault assignment in non-severe crashes is as reliable as that in severe crashes, scenario 4 can be expected to present the best results. However, limited with data, scenario 3 was considered as the best definition of high-risk for crash-involved drivers. 

#### 3.6.2. Best Model for Scenario 3 

**Figure 6:**
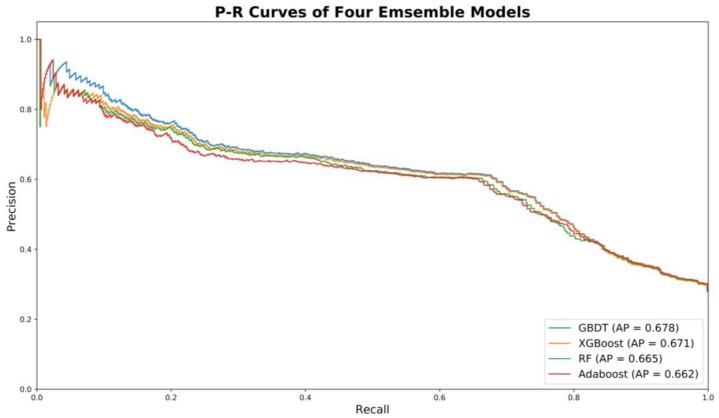
Precision–recall curves of four trained models for 2016–2017 period.

For scenario 3, among the four machine learning models, GBDT was found to have the best performance (i.e., the highest AP) on testing datasets for all time periods: 0.66, 0.65, 0.65, and 0.68. It should be noted that the performances of four machine learning techniques were very comparable. The precision–recall curves of the four candidate models (i.e., RF, Adaboost, GBDT, and XGboost) for the 2016–2017 period are shown in Figure 6. In general, for all four models, precision scores gradually decrease with the increase of recall. The precision is still around 0.60 with a recall over 0.7. Such trends indicate the trained models are relatively stable and robust. It should be noted that at the initial stage, there are some fluctuations observed for all models. In other words, predicted high-risk drivers were not observed as at-risk based on actual crash records. This could be due to the random nature of crash occurrence.

To further examine precision/recall scores at different model thresholds, a confusion matrix of the GBDT model was also developed (Table 3). There were 381 HR drivers and 37,486 NHR drivers in the testing dataset. To achieve higher precision, the model threshold can be adjusted, with the compromise of getting a lower recall. According to the Kunshan traffic police, a relatively high model prediction accuracy with an acceptable recall score can be implemented in practice. 

#### 3.6.3. Feature Importance and Partial Dependence

**Figure 7:**
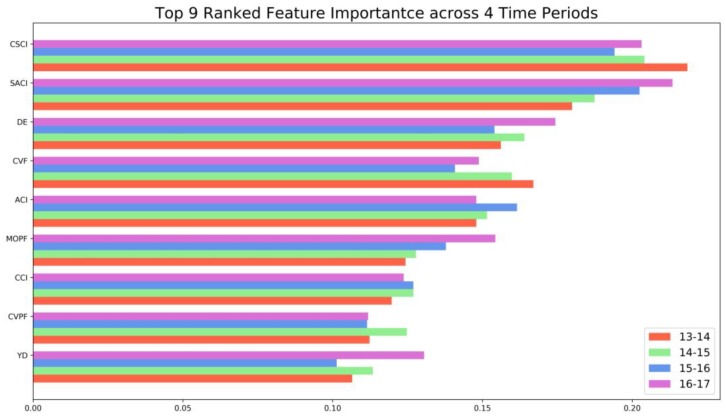
Feature importance (Gini index).

Figure 7 ranks the importance of the top nine features of four GBDT models (i.e., 2013–2014, 2014–2015, 2015–2016, 2016–2017). In general, features with the greatest importance remain the same across different periods. Note that for different time periods there were slight changes in feature importance, indicating the existence of heterogeneous temporal effects of those features. Among the top nine features, seven are all related to prior crash/violation records. The top three features are cumulative severe crash involvement (CSCI), severe at-fault crash involvement (SACI), and cumulative violation frequency (CVF), which all describe the prior crash/violation information of drivers. Other important features include at-fault crash involvement (ACI), maximum one-time penalty fee (MOPF), cumulative crash involvement (CCI), and cumulative violation penalty fee (CVPF). Overall, it appeared that crash features had more importance than violation features. This finding was consistent with previous literature that prior crash information could be more informative than violation information [11]. However, in this study, violation features were also determined to be very important. These features were rarely discussed in previous literature. Driving experience (DE) and young drivers (YD) are two important features related to driver demographics. Driving experience has already been found to affect driving risk for Chinese drivers [32]. Similarly, young drivers were often considered as an at-risk group in previous literature [33].

**Figure 8:**
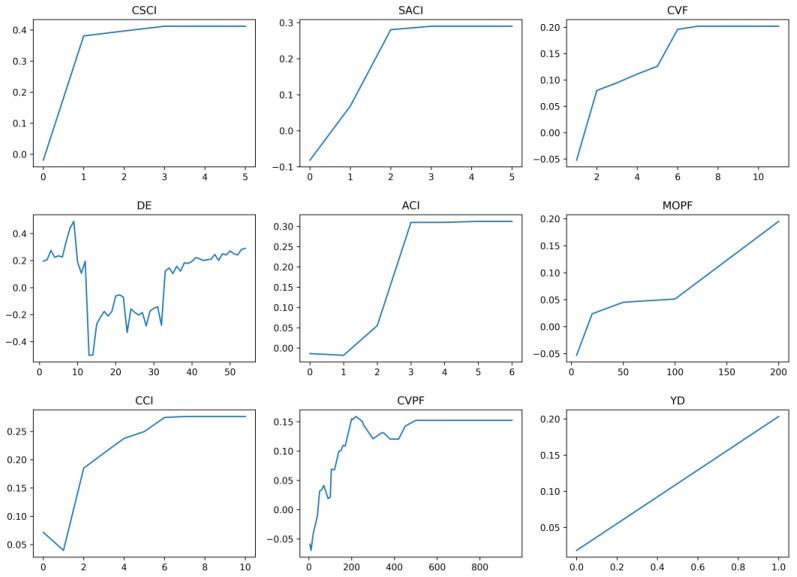
Partial dependence of top 9 important features on risk pattern.

The partial dependence of the top nine features was shown in Figure 7. Partial dependence was used to capture the relationship between features and response variable for complex models [34].

(3) $$f_{x_{i}}\left(x_{i}\right)=E_{x_{-i}}\left[f\left(x_{i},x_{C}\right)\right]=\int f\left(x_{i},x_{C}\right)d\mathbb{P}\left(x_{C}\right)$$

where $x_{i}$ is the feature *i* for which the partial dependence function needs to be calculated, $x_{C}$ are other features used in the model *f*. The partial function is estimated by calculating averages in the training data, based on the Monte Carol method:

(4) $$f_{x_{i}}\left(x_{i}\right)=\frac{1}{n}\overset{n}{\underset{i=1}{\sum }}f\left(x_{i},x_{Ci}\right)$$

where $x_{Ci}$ are actual values of feature set $x_{C}$ according to the dataset, and *n* is the number of instances in the dataset. As shown in Figure 8, for all of the top nine features, their partial dependences appear to be reasonable. Moreover, many features had non-linear relationships with high risk. For example, with the increased prior crash involvement (e.g., CSCI, SACI, ACI, and CCI), drivers’ crash risk increases significantly. As for violations, there is a general trend that driving risk increases with the increment of violation frequency (CVF) and penalty fee (MOPF and CVPF). However, such magnitude is not as large as that of crash features. To note, there is an approximate ‘U’ shape observed for driving experience (DE). Thus, models based on simple linear assumptions could easily cause bias and result in low model performance, as reported in previous literature. 

### 3.7. Model Transferability Tests

**Figure 9:**
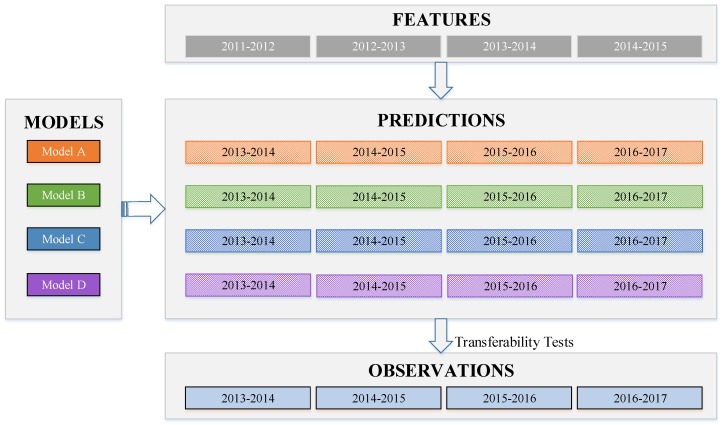
Temporal transferability tests of four trained models

It is known that drivers’ risk is time-varying, with their change in age, driving experience, social-economic status, physical and mental health, and driving skills. Thus, a previously trained model needs to be proved as temporally transferable, so that it can be used to predict drivers’ future crash risk. For example, the 2016–2017 model (i.e., Model D) was developed to capture the risk pattern between 2016 and 2017. In practice, it is questionable that this model can be directly used to predict potential high-risk drivers in 2018–19. Figure 9 illustrates the temporal transferability tests of all models for four time periods. The basic idea was that using a trained model to predict HR drivers in other time periods. For example, Model D was used to predict HR drivers for 2013–2014, 2014–2015, and 2015–2016 period. Those predictions were then compared to actual observations to examine model transferability. It was expected that drivers’ risk was time-varying during relatively longer time periods, but stable within relatively shorter time periods. Since there were four models and four time periods, the AP of each model on each time period was calculated.

**Figure 10:**
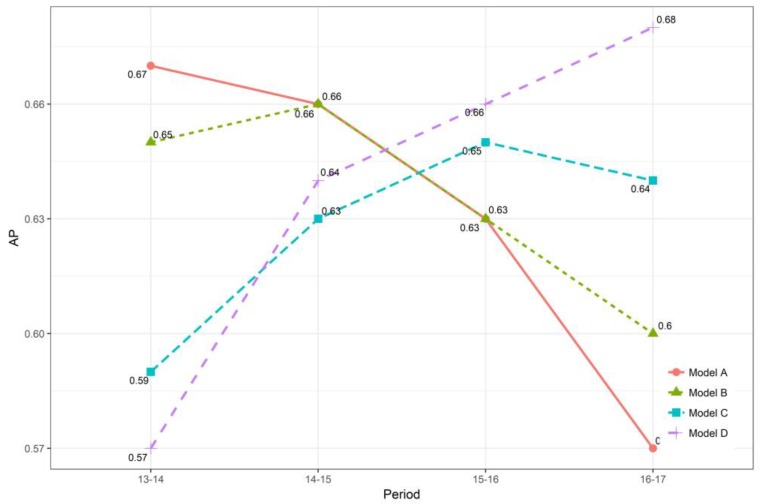
Temporal transferability results.

The results are shown in Figure 10. The findings are two-fold. First, for each time period, the pre-trained model appears to be better than other models, with the highest AP. When using a pre-trained model to predict the HR drivers in other time periods, the model performance will decrease. Moreover, it appears that such a trend is non-linear with the increase of time intervals. For instance, when predicting the 2014–2015 period, the performance of Model A has a slight decrease in AP from 0.67 to 0.66. However, when predicting HR drivers in 2015–2016 and 2016–2017 time periods, AP largely declines. Similar results were also found for Model B, C, and D. This indicates the potential time effect on drivers’ risk pattern. Thus, in practice, it is better to constantly update a trained model to keep its best performance with a time-window constraint.

Second, within relatively shorter time periods (e.g., one- or two-year time interval), model performances remain relatively stable and the temporal transferability holds. This guarantees that high-risk drivers in subsequent short periods can be predicted, using the most updated model based on previous years.

## 4. A Follow-up Study

A follow-up study was conducted to further examine the model transferability. Using the most updated model (i.e., Model D), the number of high-risk drivers in 2018–2019 was predicted. The threshold for determining high risk was set to 0.584, which achieved a precision of 0.706 on the 2016–2017 testing set. Until June 2018, half-year crash records were collected. For the top 100 HR drivers, 20 had already been involved in a crash. Thus, the expected two-year crash involvement of top 100 HR drivers could be 20*4 = 80. However, this was not guaranteed for a relatively short observation period (i.e., half a year). Since the observation period is short, the major intent of this study was to compare the risk of the two predicted groups (HR versus NHR) in 2018–19. The relative risk can be calculated as:

(5) $$\mathrm{RR}=\frac{actual\;observation\;in\;HR/Total\;predicted\;number\;in\;HR\;}{Actual\;observation\;in\;NHR/total\;predicted\;number\;in\;NHR\;}$$

where average observation can be crash count, fault assignment, and damage cost. Based on the prediction, there were 2899 HR drivers expected in 2018–19. Until June 2018, 421 drivers had been already successfully identified with crash involvement. On the other hand, 12,217 drivers were found involved in crashes among 384,937 NHR drivers. The relative crash risk is 4.57. For crashes with major/full fault assignment, the relative risk was 12.49. For drivers with severe crash involvement, the risk ratios were even larger (20.97 and 62.77). In addition, the average property damage cost for HR drivers is 9.87 times higher than that for NHR drivers. Table 4 presents the detailed results. 

## 5. Discussion

There are several interesting findings according to the results. Regarding high-risk driver definition, scenario 3 (i.e., drivers with recurrent crash involvement or found as at-fault in severe crashes) was found as the best. Since a crash can be caused by multiple factors and randomness, drivers’ risk may not be reflected by any single dimension. For example, if a driver had two crashes during the past two years, they may be defined as high risk due to crash recurrence. However, when looking into the data, the driver was determined as non-at-fault in both crashes. Thus, it is difficult to conclude whether this driver is high risk or not. Our result was also consistent with the knowledge that driving risk should be measured from multiple aspects, including crash recurrence, severity, and fault commitment. 

As for features, seven of the nine top features were related to risky driving behaviors. It implied that risky driving features were very important for risk pattern identification, which need to be carefully extracted from raw data. For example, maximum one-time penalty fee (MOPF) and cumulative violation penalty fee (CVPF) have been rarely discussed in previous literature. However, these two features appeared to be very important according to the result. The non-linear relationships between top features and driving risk also proved the complexity of risk pattern.

The proposed systematic machine learning method achieved better prediction accuracy, compared to reported prediction results. The sampling methods indicated the potential impacts of extremely unbalanced data on prediction performances. This issue has been rarely considered in previous literature. Moreover, the average precision (AP) of the models meet the practical needs, according to the local police. With a careful threshold setting, the current model present 70.6% precision on testing dataset, which is much higher compared to previous literature (e.g., 26% for crash involvement prediction; 60% for at-fault prediction, etc.). Thus, it is necessary to apply advanced data mining techniques to identify risk patterns. More importantly, those complex relationships deserve future in-depth investigations. However, this topic was rarely discussed in previous literature. Model transferability within relatively short time intervals were also proved. However, with the increase of time interval, model performance had relatively large decreases. From the follow-up study, it has shown that the most recent model performed acceptably in predicting future high-risk drivers. This proves the validity of the risk prediction model in over a short time period. 

### Issues and Limitations

Some issues also need to be addressed. First, some may argue that only crash-involved drivers were examined while other non-crash-involved drivers were not considered. However, focusing on crash-involved drivers is important and has provided meaningful results. Practically, the local police were concerned about drivers with prior crash records and wanted to focus on this group. Theoretically, crash-involved drivers have been found to be at higher risk than non-crash-involved drivers [3]. Admittedly, the risk pattern of all drivers deserves research efforts. Second, the definition of high-risk drivers could be further examined. According to our research results, the model performance could vary significantly depending on different definitions of high-risk drivers. Notably, the definition of high risk can also vary in different countries, considering culture differences. Last but not least, there are still some issues for model training, update, and validation. Although the machine learning techniques (inherently ensemble methods) used in this paper have shown their strength, other advanced statistical methods [35,36,37] and ensemble learning strategies (e.g., stacking) could be further attempted. In addition, various sampling methods and time-window effects still deserve further in-depth investigation. 

## 6. Conclusions

Drivers with excessive crash risk need to be identified and interventions must be applied to mitigate the risk. A systematic machine learning based approach was proposed to capture the complex risk pattern of crash-involved drivers in Kunshan, China. The following major conclusions can be drawn:

(1) Driving risk is necessarily measured from multiple aspects, including frequency, severity, and fault commitment. 

(2) Detailed crash/violation features need to be considered to better reflect drivers’ prior risky driving habits, which were found to highly correlate with their future driving risk.

(3) To capture the complex driving risk pattern, the development of a systematic machine learning approach is necessary. The major advantage is to better identify the non-linear relationship between factors and crash risk, which is not easily measured in the traditional statistical methods. Explicable methods, such as tree-based ensemble methods, are highly recommended.

In general, the proposed method appears to be a promising and reliable tool to identify complex crash risk pattern. As such, policy-makers can propose/design possible interventions: 1. commercial vehicles/heavy vehicle drivers with future high risk can be monitored, by in-vehicle warning system or in future connected vehicle environment [38]; 2. safety messages can be pushed routinely to high-risk drivers; 3. drivers with potentially high risk can be asked to attend traffic school or pass exams.

## Figures and Tables

**Table 1:** Basic statistics of high-risk/non-high-risk drivers.

	Scenario 1		Scenario 2		Scenario 3		Scenario 4		Scenario 5	
	HR	NHR	HR	NHR	HR	NHR	HR	NHR	HR	NHR
**2013–2014**	2520	384316	2062	377214	3632	376504	26442	361394	39438	348398
**2014** **–** **2015**	2323	384513	2052	376784	3736	375600	41558	346278	49432	338404
**2015** **–** **2016**	2744	384092	2229	375607	3903	374753	44629	343207	54224	333612
**2016** **–** **2017**	2825	384011	2179	376057	3836	374852	50332	337504	58234	329602
**Total**	387836									

HR: High-risk drivers; NHR: Non-high-risk drivers.

**Table 2:** Final features extracted from crash/violation records.

Group	Feature	Variable	Abbreviation
Demographics	Gender	Male Driver	MAD
		Female Driver	FD
	Age	Young Driver (age < 30)	YD
		Middle-age Driver (30–65)	MD
		Older Driver (age > 65)	OD
	Car Ownership	Private Car Driver	PCD
		Bus Driver	BD
		Large Truck Driver	LTD
		Others	OE
	Driving Experience	Driving Experience	DE
	Occupation	Famer	FM
		Students	SD
		Unemployed	UE
Crash	Crash Frequency	Cumulative Crash Involvement	CCI
		Cumulative Severe Crash Involvement	CSCI
	Fault assignment	At-fault Crash Involvement	ACI
		Severe At-fault Crash Involvement	SACI
	Crash Type I	Head-on Crash Involvement	HCI
		Angle Crash Involvement	AGCI
		Sideswipe Crash Involvement	SWCI
		Rear-end Crash Involvement	RECI
		Single Crash Involvement	SCI
	Crash Type II	Collide with Pedestrians	CWP
		Collide with Motorcycles	CWM
		Collide with Cyclists	CWC
	Intoxication	Drunk Driving/Drug Driving	DD
Violation	Violation Frequency	Cumulative Violation Frequency	CVF
		Cumulative Violation Types	CTV
		Cumulative Violation Penalty Point	CVPP
	Penalty Points	Maximum One-time Penalty Point	MOPP
		Average Penalty Points per time	APP
	Penalty Fee	Cumulative Violation Penalty Fee	CVPF
		Average Violation Penalty Fee per time	AVPF
		Maximum One-time Penalty Fee	MOPF
	Dangerous Violation Counts	Red-light Running Violation	RLRV
		Traffic Sign/Markings Violation	TSMV
		Right-of-Way Violation	ROWV
		Speeding Violation over 50%	SV50
		Speeding Violation over 20–50%	SV20
		Drunk Driving Violation	DDV
		Driving with Phone Usage	DPU
		Overloading Violation	OV
	Time Period	Late Night Violation (0–6)	LNV
		Morning Peak Hour Violation (7–9)	MPHV
		Evening Peak Hour Violation (17–19)	EPHV
		Night Violation (20–24)	NV
	Location Type	Total crash >mean, severe crash >mean	TP1
		Total crash >mean, severe crash <mean	TP2
		Total crash <mean, severe crash >mean	TP3
		Total crash <mean, severe crash <mean	TP4

**Table 3:** Confusion matrix of GBDT models for the testing dataset of 2016–2017 period with different threshold.

	Predicted	Non-High-Risk	High-Risk	GBDT Model Details
Observed				
Non-high-risk		37312	174	Threshold = 0.480 Precision = 0.6; Recall = 0.685
High-risk		120	261	
Non-high-risk		37444	42	Threshold = 0.584 Precision = 0.706; Recall = 0.265
High-risk		280	101	
Non-high-risk		37472	14	Threshold = 0.646 Precision = 0.80; Recall = 0.15
High-risk		324	57	

**Table 4:** Relative risk of predicted CP over NCP between 2018.1 and 2018.6.

		Predicted # of Drivers	Total Observation	Relative Risk (HR/NHR)
Total Crash Counts	HR	2899	421	4.57
	NHR	384,937	12,217	
Total major/full fault assignment	HR	2899	326	12.49
	NHR	384,937	3465	
Severe crash involvement	HR	2899	36	20.97
	NHR	384,937	228	
Severe crash with major/full fault	HR	2899	26	62.77
	NHR	384,937	55	
Total Property Damage Estimated	HR	2899	866,546 RMB	9.87
	NHR	384,937	1,467,298 RMB	
